# Gut health in broiler chickens fed a mixture of *Hermetia illucens* and *Tenebrio molitor* meals: does it have a key role in shaping bird performance?

**DOI:** 10.1016/j.psj.2026.106966

**Published:** 2026-04-16

**Authors:** Ilaria Biasato, Talal Hassan, Davide Buzzanca, Stefano Bagatella, Achille Schiavone, Maria Teresa Capucchio, Laura Gasco, Ákos Kenéz, Ilario Ferrocino

**Affiliations:** a Department of Agricultural, Forest and Food Sciences, University of Turin, Grugliasco (TO), Italy; b Department of Veterinary Sciences, University of Turin, Grugliasco (TO), Italy; c Department of Infectious Diseases and Public Health, City University of Hong Kong, Kowloon, Hong Kong, China

**Keywords:** Broiler, Insect meal, Gut microbiome, Intestinal morphology, Feed efficiency

## Abstract

Insect meals are promising sustainable protein sources for poultry, but comprehensive insights into their effects on gut health and growth performance are lacking. This study is the first to elucidate relationships between gut health parameters and performance in broilers fed *Hermetia illucens* (HI) and *Tenebrio molitor* (TM) meals at 5% or 10% inclusion levels, singly or in a 1:1 combination. A 37-day trial used 420 male Ross 308 chicks randomly allocated to seven treatments: control (C), HI5 (5% HI), HI10 (10% HI), TM5 (5% TM), TM10 (10% TM), MIX5 (5% MIX), and MIX10 (10% MIX). By integrating intestinal histomorphometry, mucin histochemistry, multi-organ histopathology, and multi-omics cecal microbiome characterization, we identified key structural, microbial, and metabolic biomarkers associated with performance variations (false discovery rate [FDR]<0.05 and *P* < 0.05 for microbiome and histomorphology, respectively). High-performing groups (MIX5, TM5) showed microbiomes enriched in short-chain fatty acid-producing bacteria (*Veillonellaceae, Butyricicoccus, Limosilactobacillus crispatus*), positively correlated with ADG and ADFI (FDR<0.05) and negatively correlated with FCR (FDR<0.05). Favorable metabolomic profiles (increased dopamine, tyramine, malic and orotic acids; reduced 3-deoxyglucosone, hexanoylcarnitine [FDR<0.05]) and preserved balance between neutral (positively correlated with BW and ADFI [*P* < 0.05]) and acidic mucins (positively correlated with BW, ADFI and ADG, and negatively correlated with FCR [*P* < 0.05]) were also observed. Low-performing groups (MIX10, HI10) displayed microbiomes dominated by *Ruminococcaceae, Alistipes*, and l-*Eubacterium* (negatively correlated with FCR and associated with purine metabolism alterations [FDR<0.05]), alongside worsened morphology (tendency for reduced villus height in MIX10 [*P* = 0.07], positively and negatively correlated with ADG and FCR, respectively [*P* < 0.05], and thinner mucosal/muscular layers [*P* < 0.05]) and decreased neutral mucins (*P* < 0.05). TM10 maintained unaffected growth performance via beneficial taxa (*Limosilactobacillus crispatus, Tyzzerella*), and reduced *Campylobacter jejuni* and antimicrobial resistance genes (FDR<0.05). Jejunal inflammation, negatively correlated with ADG (*P* < 0.05), was not influenced by dietary treatments (*P* > 0.05). In conclusion, specific taxa (*Butyricicoccus, Veillonellaceae, Limosilactobacillus crispatus*), metabolites (dopamine, tyramine, malic and orotic acids), and mucosal features (villus height, mucin composition) were identified as biomarkers of optimal performance in insect-fed broilers.

## Introduction

Kogut and Arsenault (2016) first shed light on the concept of gut health in animal production systems, including poultry, underlying its vital importance to animal growth performance. Even if there is no clear definition for gut health that encompasses all its physiological and functional features, the relationship between intestinal barrier (comprising the microbiome, mucus layer, host-derived antimicrobial compounds, epithelium, and underlying immune tissue) and diet seems to exert a key role in shaping it (Broom, 2018). In particular, the gut microbiome provides several health benefits to the host, including supporting nutrient digestion and absorption, strengthening the intestinal epithelial barrier, promoting the development and function of the immune system, and inhibiting the proliferation of harmful pathogens by competing for space and resources (Kogut and Arsenault, 2016). In the past few decades, broiler performance has therefore been increasingly optimized through a systems-based approach that integrates diet formulation strategies and in-depth characterization of the bird gut microbiome (Dittoe et al., 2022). As optimizing feed efficiency is a crucial economic and production goal in broiler farming, recent research has increasingly focused on the gut microbiota as a potential contributor to nutrient absorption and conversion into body mass. Interestingly, high- and low-efficiency broilers seem to differ in the relative abundance of specific bacteria, such as *Bacteroides* and *Lactobacillus* (Huang et al., 2021; Liu et al., 2021), but taxonomic composition alone has not yet been conclusive (Dittoe et al., 2022). Therefore, investigating both the functional and metabolic activities of the gut microbiota through a multi-omics approach, integrating metagenomics and metabolomics to assess microbial function and metabolite production, respectively, appears to be fundamental for clearly outlining its influence on bird growth performance. This method addresses current gaps by revealing mechanistic links between microbiome dynamics and productive outcomes that taxonomy alone cannot provide. However, a holistic evaluation of gut health must extend beyond the microbiome to encompass intestinal morphology, mucosal barrier integrity, and systemic immunological homeostasis. The comprehensive characterization of the gut health requires an integrated analysis of structural parameters, including villi and crypts morphological development (Sakamoto et al., 2000; Awad et al., 2009; Laudadio et al., 2012; Rysman et al., 2023), evaluation of neutral and acidic mucin secretory dynamics that regulate pathogen adherence and nutrient permeability (Tsirtsikos et al., 2012; Biasato et al., 2020a), and assessment of histopathological alterations in metabolic and lymphoid organs, including the liver, spleen, and bursa of Fabricius (Biasato et al., 2018). Among these, parameters such as villus height (Vh) and the villus height-to-crypt depth ratio (Vh/Cd) correlate with absorption and growth (Awad et al., 2009; Laudadio et al., 2012; Rysman et al., 2023).

As gut homeostasis is readily altered by diet, the introduction of novel alternative feed ingredients into poultry diets may be constrained by the magnitude of these changes. Insects such as *Hermetia illucens* (HI) and *Tenebrio molitor* (TM) have already been demonstrated to be viable feed ingredients for broiler chickens, but their use as insect meal has also posed notable nutritional challenges (Biasato et al., 2023). Indeed, even if chitin represents an excellent fermentation substrate – thereby shaping gut microbiome and barrier integrity via short-chain fatty acids (SCFAs) production –, it may reduce nutrient digestibility when increased inclusion levels (particularly above 10%) of insect meals are adopted, leading to worsened gut health and feed efficiency (Biasato et al., 2019). Further, a negative modulation of the gut microbiota appears to be evident, as the reduction in alpha diversity, increase in potential pathogens such as *Proteobacteria* and *Helicobacter*, and decrease in potentially beneficial bacteria such as *Firmicutes* and some SCFAs-producing bacteria like *Clostridium, Coprococcus,* and *Ruminococcus* represent key findings in insect-fed broilers (Biasato et al., 2019, Biasato et al., 2020a, Biasato et al., 2020b). Furthermore, detrimental effects on intestinal histomorphometry, including decreased Vh, increased crypt depth (Cd), and reduced Vh/Cd, potentially impairing absorptive capacity and compromising growth performance, have also been highlighted in broilers and laying hens fed HI-based diets (Biasato et al., 2018; Cutrignelli et al., 2018; Dabbou et al., 2018). However, despite these findings, metagenomics and metabolomics investigations of the gastrointestinal tract in poultry fed insect-containing diets remain very limited. The most comprehensive study to date, conducted in laying hens, identified significant associations among chitinolytic bacterial species, chitinolytic enzymes, and SCFAs production, specifically propionate and butyrate (Borrelli et al., 2017). These gut microbiome alterations coincided with significant changes in productive performance, including improved feed efficiency but concurrent deterioration in lay percentage, feed intake, average egg weight, and egg mass (Marono et al., 2017). However, the authors did not investigate potential gut microbiome biomarkers that could mechanistically explain the observed variations in productive response (Borrelli et al., 2017).

To address the above-mentioned digestibility issues associated with single-species insect meals, Biasato et al. (2025) recently proposed introducing a mixture of HI and TM meals into broiler chicken diets as a novel diet formulation strategy. Indeed, using a mix of insect meals in poultry diets can boost the insect industry’s competitiveness, potentially lower single-species side effects on nutrient digestibility and meat fatty acid profiles, and possibly create synergistic benefits, as Hartinger et al. (2022) have previously identified improved fat digestibility in broiler chicks when HI meal was fed along with HI fat. However, Biasato et al. (2025) did not observe significant synergistic effects of the HI–TM mixture on growth performance when compared to single-species meals, suggesting that the productive response was mainly driven by the inclusion level rather than by insect species or their combination. Nevertheless, this experimental framework provides a valuable opportunity to apply multi-omics approaches to explore whether species-specific or dose-dependent modulations of the gut ecosystem may explain the observed performance differences, even when these are not evident at the zootechnical level.

Based on the above-described scientific rationale, the present study aims to investigate the relationships among comprehensive gut health parameters and growth performance in broiler chickens fed diets containing HI and TM meals either as single ingredients or in combination. This objective is addressed through an integrated analytical approach encompassing intestinal histomorphometry, mucin histochemistry, multi-organ histopathology, and gut microbiome analyses via multi-omics approaches (metagenomics and metabolomics). We hypothesized that lower inclusion levels (5%) of insect meals will promote cecal microbiome signatures enriched in SCFAs-producing taxa, preserve intestinal morphological integrity and mucin balance, and positively correlate with growth performance, whereas higher inclusion levels (10%) will compromise gut ecosystem functionality through chitin-mediated reductions in nutrient digestibility and associated dysbiotic shifts, leading to compromised growth performance.

## Materials and methods

### Birds and experimental design

The present study is part of ongoing research aimed at investigating the comprehensive effects of a mixture of HI and TM in broiler chickens (Biasato et al., 2025). To avoid unnecessary repetition of previously published data, a brief summary of the experimental trial is provided below. A 37-day growth trial was conducted at the commercial poultry farm “Azienda Pozzo” (Riva presso Chieri, Turin, Italy), in accordance with the European Directive 2010/63/EU on animal care and use for scientific purposes. The protocol was approved by the Ethical Committee of the University of Turin (Prot. n. 15735). A total of 420 newly hatched male Ross 308 broiler chicks were randomly assigned to 42 stainless steel pens (1.00 × 1.00 × 1.00 m), each housing 10 birds and equipped with feeders, nipple drinkers, and rice hull litter. Chicks were vaccinated at hatch against Newcastle and Gumboro diseases (subcutaneously), infectious bronchitis (ocularly), and coccidiosis (spray). The poultry house featured automatic ventilation, humidification, and feeding systems. Lighting was scheduled at 23 h light:1 h dark until day 7, then 18 h light:6 h dark until slaughter. Environmental temperature was set at 32–33°C during the first week and gradually reduced to 20–21°C by the end of the trial, while relative humidity was maintained between 60 and 70%. The stocking density was chosen not to exceed 33 kg/m^2^ at the end of the experimental trial (maintained 20-26 kg/m^2^ on average). No antimicrobials, anticoccidials, or growth promoters were administered at any point during the experimental period.

Seven isonitrogenous and isoenergetic diets were tested (6 replicates per treatment): a control diet without insect meal (C); diets containing 5% or 10% *Hermetia illucens* meal (HI5, HI10); 5% or 10% *Tenebrio molitor* meal (TM5, TM10); and 5% or 10% of a 1:1 HI and TM mixture (MIX5, MIX10), replacing soybean meal partially. Each diet was provided in three phases: starter (d 0–10, crumbled), grower (d 11–25, pelleted), and finisher (d 26–37, pelleted). All formulations met or exceeded Aviagen (2022) nutritional requirements and included 0.5% TiO₂ as an indigestible marker for digestibility assessment. Feed and water were available *ad libitum*. Bird health and mortality were monitored daily throughout the trial. Growth performance was periodically assessed throughout the experimental trial. The BW was recorded at an individual level on d 10, 25, and 37 (feed change). The ADG, the ADFI, and the feed conversion ratio (FCR) were determined at the pen level for each feeding phase (d 0–10, 11–25, and 26–37) and for the overall experimental period (d 0–37) (Biasato et al., 2025).

### Cecal content amplicon and shotgun metagenomics sequencing

On d 38 (end of the growth trial), a total of 12 birds/diet (2 animals/replicate) were selected according to the average final BW of each pen and slaughtered at a commercial abattoir according to the standard EU regulations. Cecal content was collected from both ceca apices into 2 mL sterile Eppendorf tubes, which were promptly refrigerated (for a maximum of 2 h) and frozen at −80°C until microbiome analyses. Amplicon sequencing and metabolomics analyses were performed on all 12 samples, whereas shotgun metagenomics sequencing was performed on a subset of 5 birds per diet. The reduced sample size for shotgun metagenomics (*n* = 5 per diet) reflects the substantially greater sequencing depth per sample generated by whole-genome sequencing when compared to amplicon-based approaches, which provides comprehensive functional and taxonomic resolution sufficient for differential abundance and pathway analyses. This sample size was consistent with comparable poultry metagenomics studies (Borrelli et al., 2017). The five birds per diet were randomly selected from the 12 amplicon-sequenced birds. All the shotgun libraries were prepared and sequenced in a single batch to minimize technical batch effects.

Total DNA from cecal samples was extracted using the DNeasy 96 PowerSoil Pro with the QIAcube HT workstation (Qiagen, Milan, Italy) according to the manufacturer’s instructions. Amplicon sequencing (V3-V4 region of the 16S rRNA gene, 2 × 250 bp) and whole metagenomics shotgun (2 × 150 bp) were performed by Novogene (Munich, Germany). Amplicon sequencing data were analyzed by QIIME2 software v. 1.9 (Bolyen et al., 2019). Amplicon Sequence Variants (ASVs) obtained by the DADA2 algorithm (Callahan et al., 2016) were mapped against the Greengenes2 database (McDonald et al., 2024).

Tools for shotgun metagenomic read analysis were used with default settings unless otherwise specified. Raw sequence reads were quality filtered with AdapterRemoval ver. 2.3.3 (min. quality 30) (Schubert et al., 2016), while chicken reads were removed with Bowtie2 ver. 2.5.3 using reference genome of *Gallus gallus domesticum* (Langmead and Salzberg, 2012; https://www.ncbi.nlm.nih.gov/datasets/genome/GCF_016699485.2/). The number of reads corresponding to bacterial species in the samples was obtained using Metaphlan3 ver. 3.0 (Beghini et al., 2021), while Super-focus ver. 1.4.1 (DB_98) was used for functional prediction (Silva et al., 2016). The assemblies were obtained using SPAdes ver. 3.15.5 (meta option; min. contigs length 500) (Bankevich et al., 2012), and the quality control was performed with Quast ver. 5.2.0 (Gurevich et al., 2013). Genome reconstruction was performed with Metabat2 after contig mapping on the reads with Bowtie2 (Kang et al., 2019), while CheckM ver. 1.2.2 was used to check MAGs completeness and contamination, maintaining high and medium quality bins (>90% completeness, <5% contamination (Parks et al., 2015). PhyloPhlAn ver. 3.1 was used to identify bin taxonomy (Asnicar et al., 2020), while the presence of antimicrobial resistance-related genes was evaluated on contigs using amrfinder ver. 3.10.24 (Feldgarden et al., 2019). Bowtie2 was used to compare the CARD 2020 database with the reads (Langmead and Salzberg, 2012; Alcock et al., 2020). Tormes ver. 1.3.0 was used for bins pangenome analysis of MAGs to obtain information about core/accessory genes and presence of antibiotic resistance genes (Quijada et al., 2019). Average nucleotide identity (ANI) was calculated using ANIclustermap ver. 1.3.0 (https://github.com/moshi4/ANIclustermap). The shotgun metagenomics raw sequences reads are available at the bioproject accession number PRJNA1173564 (https://www.ncbi.nlm.nih.gov/sra/PRJNA1173564).

### Metabolomics analysis

The cecal content samples were subjected to targeted quantitative metabolomics analysis using the MEGA Assay of The Metabolomics Innovation Centre (TMIC; Edmonton, Canada). This assay was designed for the identification and quantification of up to 692 pre-selected metabolites including organic acids, amino acids and their derivatives, acylcarnitines, biogenic amines, phosphatidylcholines, nucleotides and nucleosides, indoles and their derivatives, sphingolipids, sugars, phosphatidylcholines, sphingolipids, ceramides, cholesterol esters, diacylglycerols, and triacylglycerols. The analytical procedures were carried out in the laboratories of TMIC according to their standard protocol, as published previously (Zhang et al., 2024). Briefly, after metabolite extraction and phenyl-isothiocyanate (PITC)-based derivatization, the liquid chromatography - tandem mass spectrometry (LC-MS/MS) analysis was performed using an ABSciex 5500 QTrap® MS (Applied Biosystems/MDS Sciex) coupled to an UHPLC system using multiple reaction monitoring (MRM) pairs in the presence of internal standards. Separation was based on a C18 column. Solvents were 0.2% formic acid in water (A) and acetonitrile (B) with a gradient (0–95% B over 5.5 min, 500 μL/min, 50°C). Direct flow injection-MS/MS employed direct infusion (30–200 μL/min) with a 20 μL injection. Organic acid analysis used 0.01% formic acid in A/B (25–100% B over 6.5 min, 400 μL/min, 40°C). Data was acquired with Analyst 1.7.2 and processed in MultiQuant 3.0.3. Metabolite concentrations were reported in µM units (absolute concentrations). Metabolites with concentrations below the lower limit of detection (LOD) in more than 20% of the samples were filtered out (Bijlsma et al., 2006). Any remaining LOD values were replaced by 1/5 of the minimum values of their corresponding variables as a standard imputation technique.

### Morphometric, histopathological, and histochemical investigations

#### Gut morphometry

A standardized 5 cm segment of small intestine was collected from the mid-jejunum, specifically the region immediately proximal to Meckel’s diverticulum. The excised segment was immediately flushed with 0.9% saline to remove all the residual luminal contents before being fixed in 10% formalin. Subsequent tissue processing involved dehydration through a graded series of ethanol (70%, 80%, 95%, and 100%) followed by clearing in isoparaffin and final embedding in paraffin wax. The wax-embedded tissues were sectioned at a uniform thickness of 5 μm using a microtome. The resulting tissue sections, mounted on glass slides, were stained with Haematoxylin & Eosin (H&E) for general histological assessment. One H&E-stained slide per intestinal segment was examined by light microscopy using a Zeiss Axiophot microscope (Carl Zeiss, Oberkochen, Germany) coupled with a Nikon DS-Fi1 digital camera (Nikon Corporation, Tokyo, Japan) employing a 2.5X objective lens. Images were captured using NIS-Elements F software, and morphometric analysis was performed using Image-Pro Plus software (version 6.0, Media Cybernetics, Maryland, USA) (Colombiono et al., 2023). The analysis included measurements of Vh (from the tip of the villus to its base), Cd, villus width (Vw, measured at the apex, middle, and bottom portions of the villus), mucosal layer thickness, submucosal layer thickness, and muscular layer thickness. Measurements of all layer thicknesses were conducted at five standardized points within the gut layers for each captured field (Colombino et al., 2023). Ten well-oriented, intact villi with the corresponding crypts were selected for measurement on each slide. The villus surface area (VSA) was calculated using the formula proposed by Sakamoto et al. (2000): [(2π) × (Vw/2) × (Vh)], where Vw = villus width; Vh = villus height.

#### Organ histopathology

In addition to the jejunal segment of the gut, standardized sampling points were maintained across all organs: the left lobe of the liver was collected, and a transverse section was obtained from the middle portion of the spleen and the bursa of Fabricius. The following specific histopathological alterations were evaluated: gut inflammation; white pulp hyperplasia and depletion in the spleen; follicular cyst and depletion in the bursa of Fabricius; and inflammation and hepatocyte degeneration in the liver. All observed alterations were quantified using a semi-quantitative scoring system based on severity: absent (score = 0), mild (score = 1), moderate (score = 2), and severe (score = 3) (Biasato et al., 2018).

#### Gut histochemistry

For the analysis of muco-substances (mucins), additional jejunal sections were stained using specialized histochemical techniques: Periodic-Acid Schiff (PAS) staining identified neutral mucins, Alcian Blue pH 2.5 (AB) staining identified acidic sialylated mucins, and High-Iron Diamine (HID) staining identified acidic sulfated mucins. The AB, PAS, and HID staining procedures were performed according to the protocol described by Tsirtsikos et al. (2012). For each slide, ten intact villi and ten crypts were selected, and the intensity of mucin staining in goblet cells was quantified using a semi-quantitative scoring system: grade 0 for absent staining, grade 1 for mild staining, grade 2 for moderate staining, and grade 3 for marked staining. This final score was determined by both the number of positive goblet cells and the staining intensity. To ensure the integrity and objectivity of the data, all the slides were assessed by three independent observers (TH, SB, and MTC). Any cases in which initial scores were discordant among the observers were collaboratively reviewed at a multi-head microscope until a consensus was reached.

### Statistical analysis

Statistical analysis of taxonomy and genes was conducted in RStudio ver. 2023.09.1 (R ver. 4.3.2) with Microeco ver. 1.4.0 (Liu et al., 2021) and with MicrobiomeAnalyst ver. 2.0 (Lu et al., 2023). Multivariate statistics were performed in RStudio using Vegan ver. 2.6-4. The EdgeR ver. 3.42.4 and DESeq2 ver. 1.40.2 were used to obtain information about differentially abundant taxa and genes (Robinson et al., 2010; Love et al., 2014). ITol ver. 7 was then used to visualize the dendrograms (Letunic and Bork, 2024). Concentration data for cecal metabolites were analyzed in MetaboAnalyst 4.0 after log transformation and Pareto scaling (Chong et al., 2019). Principal component analysis (PCA) was then used to depict how variation in the data was distributed across the dietary treatments. The first two principal components (PC1 and PC2), which collectively explained 33.3% of total variance, were used for downstream PERMANOVA testing (based on 999 permutations, *P* < 0.05 was considered significant). Results are reported as R² (proportion of variance in the PC space explained by the grouping factor), and permutation-derived *P*-value. One-way ANOVA (post hoc: Tukey) was used to compare metabolite levels across diets (false discovery rate [FDR] < 0.05 was considered significant).

Morphometric, histopathological, and histochemical findings were statistically analyzed using SPSS Statistics software (version 29.0; IBM Corp., Armonk, NY, USA). Following preliminary assessments of data distribution and variance homogeneity, utilizing the Kolmogorov-Smirnov test for normality and Levene’s test for heterogeneity, appropriate statistical models were selected. The histopathological and histochemical scores, which typically violate assumptions for parametric analysis, were analyzed using the non-parametric Kruskal-Wallis H test (post hoc test: Dunn’s Multiple Comparison test). Conversely, intestinal morphometric data were analyzed via one-way ANOVA (post hoc test: Tukey's honest significant difference [HSD]). The results were expressed as the mean and pooled SEM. *P*-values≤0.05 were considered statistically significant.

## Results

To establish a relationship between bird growth and gut health parameters, finisher and overall growth performance were herein considered, as reported by Biasato et al. (2025). Briefly, three performance phenotypes were identified across the finisher and overall trial periods. The TM5 and MIX5 groups were the highest-performing, achieving the greatest ADG and the most efficient FCR (*P* < 0.001). The HI10 and MIX10 groups displayed the poorest outcomes, characterized by reduced BW and ADG, and the highest FCR (*P* < 0.001). The C, HI5, and TM10 groups showed intermediate performance, with the TM10 maintaining FCR comparable to the high-performing groups and the HI5 exhibiting intermediate FCR values (*P* < 0.001). The ADFI was generally the highest in the TM5, HI5, and MIX5 birds, while the HI10 showed the lowest feed consumption (*P* < 0.001). Full performance data and statistical details are reported in Biasato et al. (2025).

### Cecal microbiota

**Fig. 1:**
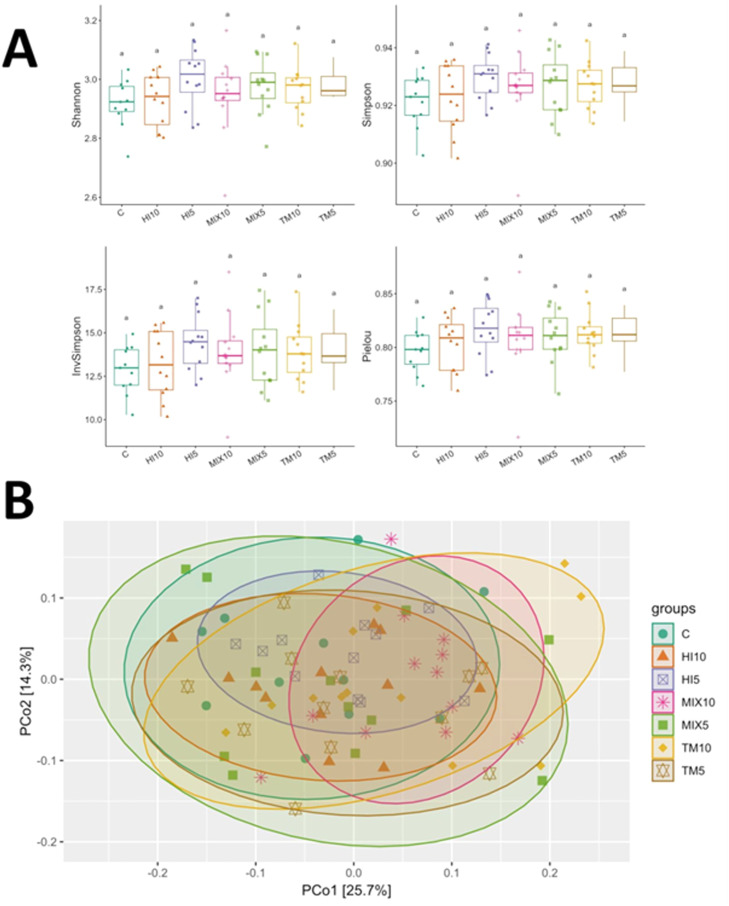
Microbial diversity in cecal content samples of broilers fed either a control diet or diets containing 5% or 10% *Tenebrio molitor* (TM5, TM10), *Hermetia illucens* (HI5, HI10), or the mix of both (MIX5, MIX10). (A) Alpha-diversity indices (Shannon, Simpson, Inverse Simpson, and Pielou’s evenness) visualized as boxplots across dietary groups. (B) Beta-diversity visualized by principal coordinate analysis (PCoA) based on Bray-Curtis distance.

**Fig. 2:**
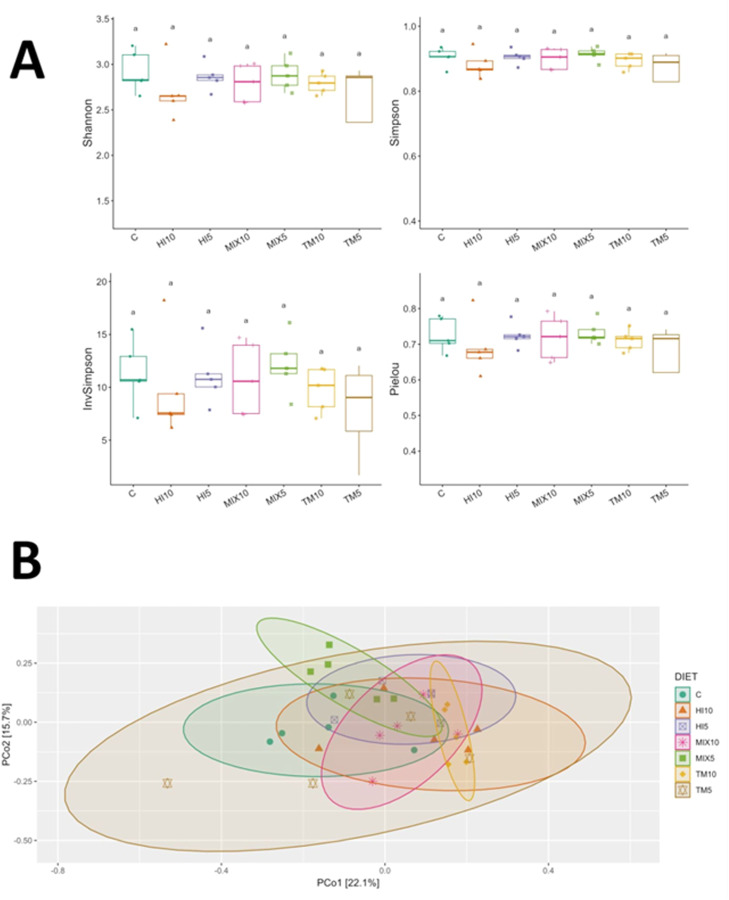
Microbial diversity based on shotgun metagenomics in cecal content samples of broilers fed either a control diet or diets containing 5% or 10% *Tenebrio molitor* (TM5, TM10), *Hermetia illucens* (HI5, HI10), or the mix of both (MIX5, MIX10). (A) Alpha-diversity indices (Shannon, Simpson, Inverse Simpson, and Pielou’s evenness) visualized as boxplots across dietary groups. (B) Beta-diversity visualized by principal coordinate analysis (PCoA) based on Bray-Curtis distances.

A total of 42 bacterial taxa were detected using amplicon sequencing (Table S1). The α-diversity indices did not differ among the dietary treatments (Fig. 1A), while β-diversity demonstrated differences in terms of Bray-Curtis distances when considering the MIX10 birds in comparison with C and HI5 groups (analysis of similarities [ANOSIM], false discovery rate [FDR]<0.05; Fig. 1B). Shotgun metagenomics detected 90 bacterial taxa (Table S2), with no differences in α-diversity indices among dietary treatments (Fig. 2A). Bray-Curtis’s distances were, instead, different between the C group and HI5, MIX5 and TM10 diets, with R values of 0.39, 0.41 and 0.74, respectively (ANOSIM, FDR<0.05; Fig. 2B). The MIX5 birds also demonstrated differences in comparison with TM5 (*R* = 0.38), TM10 (*R* = 0.72) and MIX10 (*R* = 0.44) diets (ANOSIM, FDR<0.05; Fig. 2B).

**Fig. 3:**
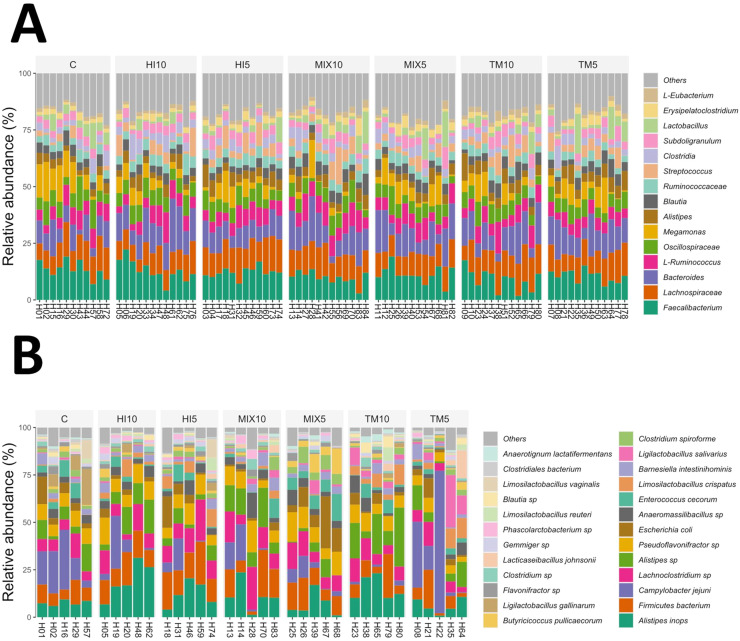
Relative abundance of dominant bacterial taxa in cecal content samples of broilers fed either a control diet or diets containing 5% or 10% *Tenebrio molitor* (TM5, TM10), *Hermetia illucens* (HI5, HI10), or the mix of both (MIX5, MIX10). (A) Stacked bar plot showing the relative abundance (%) of major bacterial taxa at the genus or family level identified by amplicon sequencing. (B) Stacked bar plot depicting the relative abundance (%) of the most abundant bacterial taxa (primarily at species or strain level) identified by shotgun metagenomics.

**Fig. 4:**
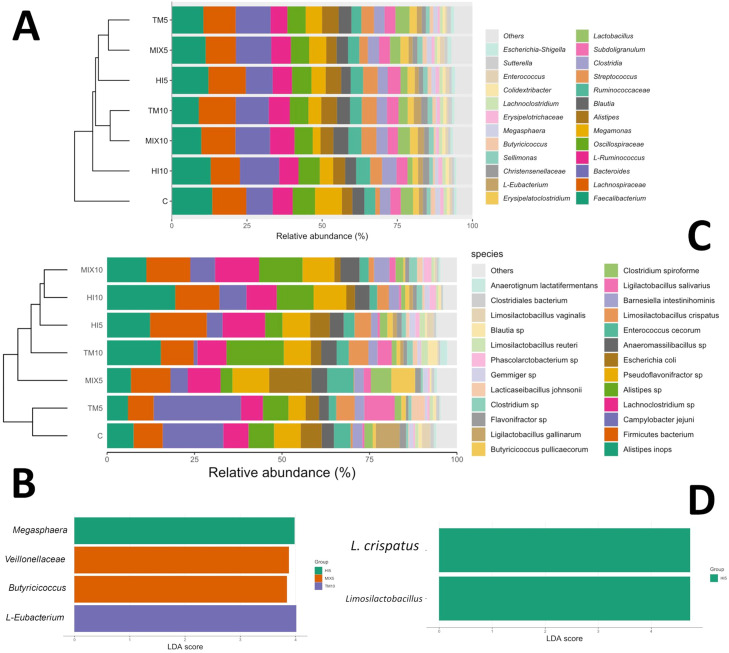
Hierarchical clustering and biomarker taxa identification in cecal microbiota of broilers fed either a control diet or diets containing 5% or 10% *Tenebrio molitor* (TM5, TM10), *Hermetia illucens* (HI5, HI10), or the mix of both (MIX5, MIX10). (A) Dendrogram (based on amplicon sequencing data) shows relative abundance of major bacterial taxa (genus/family level) across dietary groups, with hierarchical clustering revealing separation between the control (C) and insect-based diets, and further differentiation between 5% and 10% inclusion levels. (B) Linear discriminant analysis (LDA) effect size (LEfSe) plot from amplicon sequencing data highlighting discriminatory taxa (LDA score > 2, *P* < 0.05). (C) Dendrogram (based on shotgun metagenomics data) illustrates the relative abundance of bacterial species, with clustering showing no clear separation between the control (C) and TM5 groups. (D) Illustrates LDA scores; LEfSe plot from shotgun metagenomics data.

Amplicon sequencing revealed *Faecalibacterium, Lachnospiraceae, Bacteroides, Ruminococcus, Oscillospiraceae, Megamonas,* and *Alistipes* as the bacterial taxa that covered more than 50% of the relative abundance at the maximum taxa level (Fig. 3A), while *Alistipes inops, Firmicutes, Campylobacter jejuni, Alistipes* sp., and *Escherichia coli* were identified as the top five taxa detected by shotgun metagenomics (Fig. 3B). Dendrograms from amplicon sequencing showed separation between C and insect-based diets, with a further distinction between 5% and 10% inclusion levels (Fig. 4A). In contrast, shotgun metagenomic dendrograms revealed no separation between the C and TM5 birds (Fig. 4B). Linear Discriminant Analysis (LDA) on amplicon sequences showed an association between *Veilonellaceae* and *Butyricicoccus* and the MIX5 diet, while the HI5 and TM10 diets were associated with *Megasphaera* and *Eubacterium*, respectively (linear discriminant analysis effect size [LEfSe], *P* < 0.05; Fig. 4C). Differently, LDA on shotgun sequences revealed an association between *Limosilactobacillus crispatus* and the HI5 diet (LEfSe, *P* < 0.05; Fig. 4D).

Minor ASV fractions were differentially associated with the dietary treatments (Fig. 3, Fig. 4, *P* < 0.05). *Alistipes* increased in the HI5 and TM10 groups when compared to C, and in the TM5 when compared to HI10, whereas it decreased in the MIX5 diet in comparison with TM5 (*P* < 0.05). *Barnesiella* was more abundant in the MIX10 group than HI10 (*P* < 0.05). *Butyricicoccaceae* abundance increased in the MIX5 birds when compared to HI10 and TM5, whereas *Butyricicoccus* was less abundant in the HI10 and MIX10 diets than C, but more abundant in the HI5, MIX5, TM10, and TM5 birds in comparison with HI10 (*P* < 0.05). l-*Eubacterium* increased in the MIX10 group when compared to C, as well as in the TM10 diet in comparison with HI10 and HI5 (*P* < 0.05). *Megasphaera* was reduced in the MIX10 birds when compared to C, while *Ruminococcaceae* was more abundant in the MIX10 group than TM5 (*P* < 0.05). *Streptococcus* showed increased abundance in all the insect-based diets in comparison with C, while *Tyzzerella* and *Veillonellaceae* were associated with the TM10 and MIX5 birds, respectively (*P* < 0.05). Shotgun metagenomics revealed that the highest inclusion level of insect meals (10%) was positively correlated with *Alistipes* (*r* = 0.52; FDR<0.03), while the TM10 diet demonstrated a lower abundance of *Campylobacter jejuni* than C (log_2_ FC of −3.25; edgeR, FDR<0.05). A higher abundance of *Limosilactobacillus crispatus* was also highlighted in the TM5 and TM10 groups when compared to C (log_2_ FC of 2.53 and 2.29, respectively; edgeR, FDR<0.05), whereas the HI5 diet demonstrated increased abundance of *Lachnoclostridium* in comparison with C (log_2_ FC of 1.98; edgeR, FDR<0.05).

### Cecal metagenome

Shotgun metagenomics yielded 1676 metagenomics assembled genomes (MAGs). However, ANI analysis did not demonstrate a strong association between diets and MAGs, considering the top 10 most abundant bacterial species. Reads related to different EC functions resulted, however, differentially abundant when comparing the insect-based diets to C (edgeR; FDR<0.05; Tables S3 and S4). The TM5 birds displayed a higher abundance of bacteriocin ABC transporter (log_2_ FC of 4.09) and immunity protein (log_2_ FC 4.18) related genes than C (edgeR; FDR<0.05). Furthermore, diets containing 5% of insect meals demonstrated that sucrose-6-phosphate hydrolase (log_2_ FC of −7.28) and polysaccharide biosynthesis glycosyl transferase CpsN (log_2_ FC of −2.11) related genes were less abundant when compared to C (edgeR; [FDR] <0.05). Differently, diets containing 10% of insect meals showed a lower percentage of phage lysozyme (log_2_ FC of −3.97) and phage integrase family proteins (log_2_ FC of −3.30) related genes in comparison with C (edgeR; FDR<0.05).

### Cecal metabolome

**Fig. 5:**
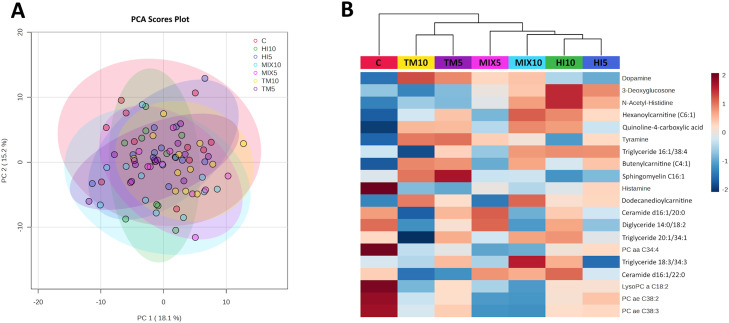
(A) Principal component analysis scores plot of all metabolites and (B) heatmap visualization of the top 20 metabolites detected by metabolomics analysis of cecal content samples of broilers fed either a control diet or diets containing 5% or 10% *Tenebrio molitor* (TM5, TM10), *Hermetia illucens* (HM5, HM10), or the mix of both (MIX5, MIX10).

**Fig. 6:**
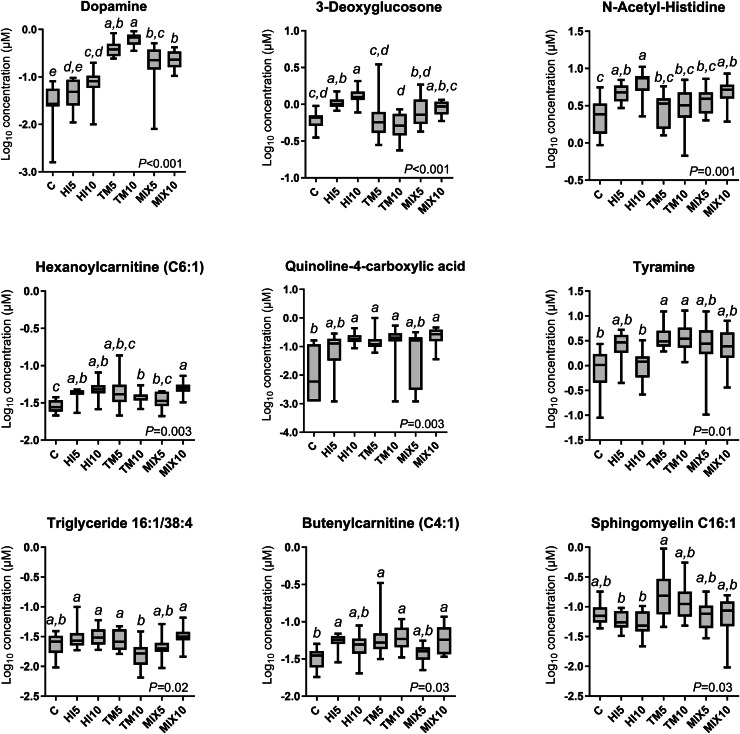
Concentrations of significant metabolites detected by ANOVA of cecal content metabolome data of broilers fed either a control diet or diets containing 5% or 10% *Tenebrio molitor* (TM5, TM10), *Hermetia illucens* (HM5, HM10), or the mix of both (MIX5, MIX10). *P*-values were subjected to false discovery rate correction. Box plots show median and interquartile range, while whiskers indicate the minimum to maximum range. Different superscripts indicate significant differences (Tukey’s post hoc test, *P* < 0.05).

After the filtration, 579 metabolites were retained (Table S7). The PCA did not identify any obvious separation among the dietary treatments (Fig. 5A), while the PERMANOVA results (R^2^=0.12, *P* = 0.055) suggested a near-significant, moderate treatment effect (i.e., dietary grouping explaining 12% of the multivariate variance captured by the first two principal components). The top 20 metabolites were visualized in a heatmap (Fig. 5B); however, only the top 9 metabolites showed significant differences among the dietary treatments (FDR<0.05, Fig. 6). In detail, the TM-fed groups showed higher dopamine levels than the C and HI diets, with the MIX groups showing intermediate values (FDR<0.001). Similarly, increased tyramine was observed in the TM-fed birds in comparison with the C and HI10 diets, while the MIX and HI5 groups displayed intermediate concentrations (FDR<0.05). The TM5 diet was also characterized by higher sphingomyelin C16:1 than HI-fed broilers, with the other groups showing intermediate values (FDR<0.05). In contrast, the HI-based diets were characterized by higher 3-deoxyglucosone levels than in C- and TM-fed birds, while the MIX groups displayed intermediate concentrations (FDR<0.001). Higher N-acetyl-histidine was also identified in the HI10 diet compared with the TM and C groups, with the other broilers showing intermediate values (FDR<0.01). Furthermore, the HI10- and MIX10-fed birds were characterized by increased hexanoylcarnitine (C6:1) in comparison with MIX5 and C diets, while the other groups displayed intermediate concentrations (FDR<0.01). The C broilers showed lower quinoline-4-carboxylic acid and butenylcarnitine (C4:1) than MIX10, TM, and HI10, and TM, MIX10, and HI5 groups, respectively (FDR<0.01 and FDR<0.05). Lastly, decreased triglyceride (TG) 16:1/38:4 was highlighted in the TM10-fed birds when compared to HI, MIX10, and TM5 groups (FDR<0.05).

### Correlation between gut microbiome and growth performance

Pearson’s correlation about amplicon sequencing data revealed that part of the taxa was associated with the cecal metabolome and growth performance of the birds (0.3 < r>−0.3, FDR<0.05; Tables S5 and S6). Acetic acid was negatively correlated with *Barnesiella*, while the latter was also positively correlated with pimelylcarnitine, 2‑hydroxy-3-methylvaleric acid, and 2-hydroxybutyric acid (FDR<0.05). Diglycerides (DG) 16:0/18:1, DG 16:0/18:2, DG 16:1/18:2, DG 18:1/18.2, DG 18:2/18.2, hexosylceramide (HexCer) d18:1/18:0, HexCer d18:1/18:1, TG 18:1/38:5, N2-acetyl-ornithine, xanthosine, malic acid, and orotic acid were positively associated with *Butyricicoccaceae*, while cytidine was negatively correlated with *Veillonellaceae* (FDR<0.05). Ceramide (Cer) d16:1/20:0 and DG 16:0/18:2 metabolites were negatively correlated with *Alistipes*, while 3-hydroxybutyric acid and 4-hydroxyphenylpyruvic acid were positively correlated with *Megasphaera* (FDR<0.05). The BW, ADG, and ADFI of the finisher phase and the ADG and ADFI of the whole experimental trial were positively correlated with *Butyricicoccus* and *Veillonellaceae*, while *Butyricicoccus* alone was also negatively correlated with the FCR of the finisher phase (FDR<0.05). *Ruminococcaceae* were negatively correlated with the ADFI and BW of the finisher phase, and the ADG and ADFI of the whole experimental trial, while this family was positively correlated with Cer d18:1/16:0, DG 16:0/18:2, DG 16:1/18:2, DG 18:1/18:1, DG 18:1/18:2, and DG 18:2/18:2 (FDR<0.05). *l-Eubacterium* displayed a negative correlation with N2-acetyl-ornithine, Cer d16:1/20:0, DG 16:0/18:2, DG 18:2/18:2, DG 18:1/18:2, and orotic acid, as well as with the ADFI of the finisher phase (FDR<0.05).

Shotgun metagenomics taxa that demonstrated differences in terms of relative abundances among the dietary treatments also displayed correlations with pathways, metabolite concentrations, and growth performance (0.5 < r > −0.5, FDR<0.05; Tables S5 and S6). *Limosilactobacillus crispatus* was positively correlated with butenylcarnitine (C4:1) and 103 Enzyme Commission (EC) numbers of related pathways, such as the biosynthesis/quorum sensing regulatory system of lantacin B, and genes related to bacteriocin-like peptide N BlpN (FDR<0.05). Moreover, carbohydrate-related reads were positively correlated with *Limosilactobacillus crispatus*, including reads related to maltose, galactose, ribose, arabinose, fructose, succinate, glucose, raffinose, and pyruvate sugars (FDR<0.05). *Campylobacter jejuni* was positively correlated with multidrug resistance RND efflux system inner membrane transporter MexD and MexF, macrolide specific efflux protein MacA, penicillin-binding protein 2 (PBP2), multidrug efflux transporter major facilitator superfamily MFS TC 2A1, broad-specificity multidrug efflux pump YkkD and beta lactamase-related reads (FDR<0.05). *Lachnoclostridium* was positively correlated with urea ABC transporter substrate binding protein (UrtA) related read, while *Alistipes* was positively correlated with fructose, fucose, and maltose metabolism, and urease accessory protein UreD, but negatively correlated with PTS system and sucrose-specific IIB component related reads (FDR<0.05).

### Morphometric, histopathological and histochemical investigations

#### Gut morphometry

The morphometric data (Table 1) indicate that incorporating insect meal altered the jejunum's physical structure in broiler chickens, affecting Vw and the overall thickness of the intestinal layers (*P* < 0.05). In contrast, the Cd, Vh/Cd, and VSA remained unchanged across all dietary treatments (*P* > 0.05). The most notable effect was observed in Vw, where dietary HI meal inclusion led to wider villi compared with the C- and TM-fed groups (*P* < 0.001). Although Vh showed an overall significant ANOVA (*P* = 0.042), the post hoc tests did not identify any specific group as different from the others (*P* > 0.05). However, Vh tended to be numerically higher in the C group than in the MIX10 diet, with a mean difference of 0.360 (*P* = 0.070). Conversely, the birds fed the MIX10 diet showed decreased mucosal layer width and muscular layer width when compared to the C and HI5 groups (*P* = 0.007 and *P* = 0.012, respectively).

**Table 1:** Jejunal morphometry of the broiler chickens fed the control and the insect-based experimental diets (*n* = 12).

Histomorphology parameters	Dietary treatments							SEM	*P-value*
	C	H5	H10	TM5	TM10	MIX5	MIX10		
Vh (mm)	1.074^a^	0.999^a^	0.759^a^	0.963^a^	0.922^a^	1.004^a^	0.715^a^	0.253	0.042
Vw (mm)	0.422^a^	0.526^b^	0.502^b^	0.408^a^	0.441^a^	0.477^ab^	0.492^b^	0.067	<0.001
Cd (mm)	0.062	0.056	0.055	0.060	0.057	0.066	0.053	0.015	0.597
Vh/Cd	23.132	26.640	21.570	21.957	23.225	22.166	21.914	5.490	0.508
VSA (mm²)	1.501	2.080	1.536	1.358	1.427	1.688	1.514	0.884	0.510
Mucosal layer width (mm)	1.513^b^	1.403^b^	1.062^ab^	1.240^ab^	1.166^ab^	1.313^ab^	0.941^a^	0.314	0.007
Muscular layer width (mm)	0.296^b^	0.226^ab^	0.268^ab^	0.266^ab^	0.244^ab^	0.315^b^	0.195^a^	0.068	0.012

Cd, crypt depth; Vh, villus height; Vh/Cd, villus height-to-crypt depth ratio; VSA, villus surface area; Vw, villus width. C, control diet; HI5, diet containing 5% of *Hermetia illucens* meal; HI10, diet containing 10% of *Hermetia illucens* meal; TM5, diet containing 5% of *Tenebrio molitor* meal; TM10, diet containing 10% of Tenebrio molitor meal; MIX5, diet containing 5% of a 1:1 mixture of *Hermetia illucens* and *Tenebrio molitor* meals; MIX10, diet containing 10% of a 1:1 mixture of *Hermetia illucens* and *Tenebrio molitor* meals.

Means with different superscript letters (a, b) within each row denote significant differences among dietary treatments (*P* ≤ 0.05).

#### Organ histopathology

The histopathological analysis showed that insect meal inclusion had no impact on any of the examined organs, including the liver, jejunum, spleen, or bursa of Fabricius (Table 2).

**Table 2:** Histopathological scores of the organs of the broiler chickens fed control and the insect-based experimental diets (*n* = 12).

Organs	Histopathology parameter	Dietary treatments							SEM	*P-value*
		C	HI5	HI10	TM5	TM10	MIX5	MIX10		
Liver	Inflammation	0.792	0.583	0.583	1.042	0.234	1.000	0.625	0.070	0.396
	Degeneration	1.500	1.500	1.375	1.458	0.222	1.417	1.917	0.083	0.693
Jejunum	Inflammation	1.083	1.375	1.500	1.625	1.125	1.375	1.667	0.083	0.457
Spleen	Follicular hyperplasia	0.458	0.182	0.250	0.250	0.115	0.083	0.182	0.042	0.644
	Follicular depletion	0.000	0.000	0.042	0.000	0.083	0.042	0.000	0.015	0.686
Bursa of Fabricius	Follicular cyst	0.227	0.450	0.083	0.542	0.444	0.600	0.545	0.066	0.186
	Follicular depletion	0.318	0.200	0.375	0.292	0.500	0.364	0.150	0.043	0.546

C, control diet; HI5, diet containing 5% of *Hermetia illucens* meal; HI10, diet containing 10% of *Hermetia illucens* meal; TM5, diet containing 5% of *Tenebrio molitor* meal; TM10, diet containing 10% of Tenebrio molitor meal; MIX5, diet containing 5% of a 1:1 mixture of *Hermetia illucens* and *Tenebrio molitor* meals; MIX10, diet containing 10% of a 1:1 mixture of *Hermetia illucens* and *Tenebrio molitor* meals.

Histopathological scores (semi-quantitative) ranged from 0 (none) to 3 (severe), with intermediate scores indicating mild (1) and moderate (2) observations. Means with different superscript letters (a, b) within each row denote significant differences among dietary treatments (*P* ≤ 0.05).

#### Gut histochemistry

The histochemical analysis showed no differences among the dietary treatments for the AB and HID staining (Table 3). In contrast, the C, TM5, and MIX5 groups showed higher villi PAS scores than the HI-based and MIX10 diets, with TM10 birds showing intermediate values (*P* = 0.038).

**Table 3:** Histochemical scores of the jejunum of the broiler chickens fed the control and the insect-based experimental diets (*n* = 12).

Stain	Histochemistry parameters	Dietary treatments							SEM	*P-value*
		C	HI5	HI10	TM5	TM10	MIX5	MIX10		
AB	Villi	1.111	0.700	0.800	1.233	0.867	1.233	0.600	0.086	0.316
	Crpts	1.028	0.792	0.750	1.000	0.667	0.806	0.889	0.079	0.709
PAS	Villi	1.444^a^	0.667^b^	0.767^b^	1.600^a^	1.133^ab^	1.300^a^	0.733^b^	0.100	0.038
	Crpts	1.333	0.694	0.583	0.933	0.806	0.889	0.722	0.096	0.354
HID	Villi	1.028	0.767	0.633	1.033	0.933	1.067	0.400	0.071	0.092
	Crpts	0.778	0.556	0.633	0.800	0.583	0.750	0.417	0.061	0.419

AB, Alcian Blue pH 2.5 staining; HID, High-Iron Diamine staining; PAS, Periodic-Acid Schiff staining; C, control diet; HI5, diet containing 5% of *Hermetia illucens* meal; HI10, diet containing 10% of *Hermetia illucens* meal; TM5, diet containing 5% of *Tenebrio molitor* meal; TM10, diet containing 10% of *Tenebrio molitor* meal; MIX5, diet containing 5% of a 1:1 mixture of *Hermetia illucens* and *Tenebrio molitor* meals; MIX10, diet containing 10% of a 1:1 mixture of *Hermetia illucens* and *Tenebrio molitor* meals.

Histochemistry scores (semi-quantitative) ranged from 0 (none) to 3 (severe), with intermediate scores indicating mild (1) and moderate (2) observations. Means with different superscript letters (a, b) within each row denote significant differences among dietary treatments (*P* ≤ 0.05).

### Correlation between histomorphometry parameters and growth performance

**Fig. 7:**
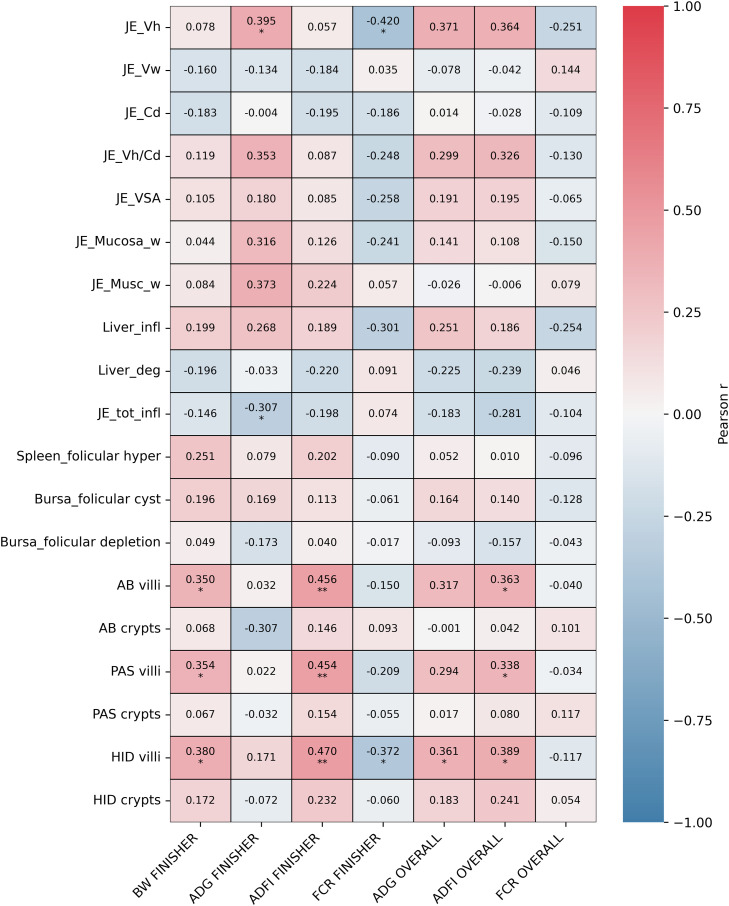
Correlation matrix of gut health parameters and production traits. The heatmap represents the relationships between jejunal morphology (JE; jejunum, Vh; villus height, Vw; villus width, Cd; crypt depth; VSA; villus surface area; Mucosa_w; width of mucosal layer, Musc_w; width of muscular layer), organ health including liver, spleen and Bursa of Fabricius (Bursa; Bursa of Fabricius, infl; inflammation, deg; degeneration, tot_infl; total inflammation, hyper; hyperplasia), mucin composition (AB; Alcian blue, PAS; Periodic Acid-Schiff, HID; High Iron Diamine), and growth metrics (BW; Body weight, ADG; Average daily gain, ADFI; Average daily feed intake, FCR; Feed conversion ratio). The color gradient represents the Pearson coefficient (r), ranging from negative 1 (blue) to positive 1 (red), with correlation coefficient values in the box. * *P* < 0.05, ** *P* < 0.01. (For interpretation of the references to color in this figure legend, the reader is referred to the web version of this article.).

Correlations between histological parameters and growth performance revealed significant associations during the finisher phase (Fig. 7). Jejunal Vh was positively correlated with the ADG during the finisher period (*r* = 0.395, *P* < 0.05) and negatively correlated with the FCR for the same feeding phase (r=−0.420, *P* < 0.05). In contrast, jejunal inflammation showed a negative correlation with finisher ADG (r =−0.307, *P* < 0.05). Regarding the mucin production in the gut, both the villi AB and PAS staining scores were positively correlated with the finisher BW (*r* = 0.350 and *r* = 0.354, respectively; *P* < 0.05), finisher ADFI (*r* = 0.456 and *r* = 0.454; *P* < 0.01), and overall ADFI (*r* = 0.363 and *r* = 0.338; *P* < 0.05). The villi HID staining showed the most extensive significant relationships, correlating positively with the finisher BW (*r* = 0.380, *P* < 0.05), finisher ADFI (*r* = 0.470, *P* < 0.01), overall ADG (*r* = 0.361, *P* < 0.05), and overall DFI (*r* = 0.389, *P* < 0.05). Furthermore, HID villi were the only mucin parameter negatively correlated with finisher FCR (*r* = −0.372, *P* < 0.05).

## Discussion

Biasato et al. (2025) recently reported that including 5% of TM and MIX meals in the diets of broiler chickens in the present study improved their growth performance, whereas the 10% inclusion level of HI and MIX meals was associated with the poorest outcomes. The 5% HI and 10% TM diets were associated with growth patterns similar to those of the feed regularly used in commercial farming. Therefore, the current research aimed to identify gut health markers associated with the observed growth performance across different dietary treatments. To this end, the host-microbiome-diet interactions are discussed according to the distinctive phenotypes, i.e., for the high-performing groups (TM5 and MIX5) vs. the low-performing groups (HI10 and MIX10) in relation to the control and the intermediate groups (HI5 and TM10), with emphasis on microbial, metabolic, and structural biomarkers.

### Gut health in high-performing broiler chickens

#### Gut microbiome

Amplicon and shotgun metagenomics sequencing and metabolomics revealed significant differences between the TM5- and MIX5-fed birds (high-performing dietary treatments) and the C, and the HI10 and MIX10 groups (reference and low-performing dietary treatments, respectively).

The cecal microbiota of MIX5 broilers was significantly distinct from those of C, MIX10, and TM5, with *Veillonellaceae* and *Butyricicoccus* associated with the MIX5 group. *Veillonellaceae* are common members of the poultry gut microbiota and comprise genera such as *Veillonella, Megamonas*, and *Megasphaera*, which produce SCFAs, including propionate and butyrate (Rychlik et al., 2020). *Butyricicoccus* is another butyric acid-producing genus that has been suggested as a potential gut marker of reduced fat deposition in broiler chickens (Liu et al., 2023), as well as being more abundant in low-residual feed intake, high-efficiency birds (Liu et al., 2021). Identification of SCFA-producing bacteria is a common finding in the gut microbiota of insect-fed birds, resulting from chitin fermentation. SCFAs have multiple benefits for gut health, as they can enhance intestinal epithelial cell barrier function by serving as an energy source for enterocytes and stimulating goblet cell differentiation and mucus production, and by reducing enteric pathogens due to their antimicrobial properties (Biasato et al., 2023). The role of SCFA-producing bacteria as a gut marker of improved growth performance is supported herein by the positive correlation between *Veillonellaceae* and *Butyricicoccus* and LW, ADG, and ADFI of the finisher phase and the ADG and ADFI of the whole experimental trial, with *Butyricicoccus* alone being also negatively correlated with the FCR of the finisher phase. Interestingly, *Veillonellaceae* was negatively correlated with cytidine, an endogenous metabolite in the pyrimidine metabolism pathway that has been shown to alleviate dyslipidemia and improve hepatic steatosis in mice by increasing the abundance of SCFA-producing gut microbiota (Niu et al., 2023). Therefore, we can hypothesize that cytidine may facilitate the proliferation of these beneficial bacteria by serving as their nitrogen source (Qu et al., 2025). However, since other SCFAs producers, such as *Subdoligranulum variabile* and *Peptostreptococcaceae*, appear to be negatively associated with feed efficiency in broiler chickens (Liu et al., 2021), other changes in the gut microbiome must be considered to explain the observed differences in growth performance. *Butyricicoccaceae*, the family of *Butyricicoccus,* was also enriched in the MIX5-fed broilers when compared to the HI10 and TM5 diets, being positively correlated with selected lipids and lipid-like molecules (diacylglycerols, hexosylceramides, and triacylglycerols), N2-acetyl-l-ornithine, xanthosine, and malic and orotic acids. Glycerolipids, such as diacylglycerols and triacylglycerols, represent one of the major lipid classes found in bacterial membranes, whereas sphingolipid molecules (e.g., hexosylceramides) are found only in some bacterial taxa (Zhang et al., 2024). The relative abundance of the metagenomic reads assigned to pathways for fatty acids, lipids, and isoprenoids was very low across all samples (0.63–1.02% of total reads), thus indicating a minimal microbial capacity for boosting metabolites such as diacylglycerols, triacylglycerols, hexosylceramides, and N2-acetyl-l-ornithine. Therefore, the observed correlations with *Butyricicoccaceae* reflect associations rather than an active microbial synthesis. While scientific evidence for these molecules in *Butyricicoccaceae* membranes is still lacking, the absence of enrichment in lipid metabolism pathways makes a major microbial contribution unlikely. However, potential host-derived sources should also be considered as alternative origins for these metabolites, highlighting the need for further studies integrating host and microbial metabolic profiling. Regardless of origin, sphingolipids from the mouse gut microbiome have previously been reported to exert a desirable anti-inflammatory effect by decreasing the number of colonic natural killer (NK) T cells (Brown et al., 2023), thereby potentially highlighting their beneficial role in gut health. N2-acetyl-l-ornithine is a metabolite of the *de novo* biosynthetic pathway for ornithine (and, therefore, arginine) in bacteria, with glutamate being used as a precursor (Nelson et al., 2021). Xanthine, of which xanthosine is a derivative, is a host-microbial co-metabolite widely found in gut microbiota that can be converted to uric acid by xanthine oxidase (Wei et al., 2021). Interestingly, cecal bacteria can catabolize uric acid to ammonia, which the host can absorb and use to synthesize glutamine, a precursor of glutamate (Pan and Yu, 2014). As other SCFA-producing bacteria (i.e., *Roseburia*) have previously been associated with both N2-acetyl-l-ornithine (Gu et al., 2024) and xanthine (Wei et al., 2021), the involvement of *Butyricicoccaceae* in their metabolism can be hypothesized herein. Both metabolites could have exerted a positive influence on the gut of the MIX5-fed broilers, as arginine can protect intestinal epithelial cells from apoptosis triggered by lipopolysaccharide-induced oxidative damage (Zhang et al., 2019), while xanthine appears to be involved in the resistance to high-fat diet-induced obesity in mice (Wei et al., 2021). Malic acid is a key metabolite in the tricarboxylic acid (TCA) cycle (Lu et al., 2025), while orotic acid is a key intermediate in the pyrimidine biosynthesis pathway (Löffler et al., 2015). Both metabolites, derived from the gut microbiota or the host, may contribute to improved feed efficiency in broilers by playing pivotal roles in mitochondrial energy production and redox homeostasis (Shimamoto et al., 2020; Jiang et al., 2025), thereby making their identification in the MIX5 group reasonable. The cecal metabolome of the MIX5 broilers was also associated with increased dopamine concentration in comparison with the C diet. Dopamine is a neurotransmitter whose bioavailability is essential for normal brain functioning, and whose concentrations are modulated via bidirectional communication known as the microbiota-gut-brain axis (Hamamah et al., 2022). Dopamine has been directly detected across all the regions of the broiler chicken intestinal tract (Lyte et al., 2021), thus confirming its enteric presence in poultry. However, while enterochromaffin cells are established producers of approximately 90% of peripheral serotonin, their specific role in enteric dopamine synthesis in poultry remains uncharacterized. The elevated cecal dopamine observed in the MIX5 and TM5 groups may plausibly reflect SCFAs-mediated signaling, as microbial SCFAs have been shown to bind receptors on enteroendocrine cells and modulate neurotransmitter release and reuptake in mammalian models (Strandwitz, 2018). However, this proposed mechanism remains a hypothesis in the present study, as enterochromaffin cell density was not directly quantified, plasma dopamine concentrations were not measured, and dopamine transporter (DAT) expression in the intestinal tissue was not assessed. Furthermore, breed-specific and age-dependent variations in enteric neurochemical metabolism have recently been documented in poultry (Wickramasuriya and Lyte, 2025), thus suggesting that the dopamine response herein observed may not be generalizable across all broiler genotypes. Future studies incorporating immunohistochemical quantification of enteroendocrine cells, plasma neurotransmitter profiling, and receptor expression analyses are needed to validate this mechanistic link. The metabolome of the MIX5 broilers was also associated with reduced 3-deoxyglucosone and hexanoylcarnitine (C6:1) concentrations in comparison with the HI10, and HI10 and MIX10 diets, respectively. 3-deoxyglucosone is an advanced glycation end‑product that is formed due to condensation between the carbonyl group of a reducing sugar and the free amine group of proteins, lipids, or nucleic acids, and that may be associated with the reduction in SCFA-producing bacteria and impaired carbohydrate and amino acid metabolism in the gut (Aschner et al., 2023). In parallel, high levels of acylcarnitines can indicate disturbances in mitochondrial fatty acid oxidation, which impair the breakdown of fats for energy production (Smith et al., 2021). Therefore, the improved growth performance of the MIX5-fed birds may also be related to increased dopamine production, potentially mediated by SCFA-producing bacteria, and a decrease in metabolites indicative of perturbations in carbohydrate and amino acid metabolism or energy production.

The cecal microbiota of the TM5 broilers displayed an increased abundance of *Limosilactobacillus crispatus* when compared to the C diet, with these taxa being positively correlated with biosynthesis/quorum-sensing regulatory system of lantacin B, bacteriocin-like peptide N BlpN, and carbohydrate (maltose, galactose, ribose, arabinose, fructose, succinate, glucose, raffinose, and pyruvate) related genes, and butenylcarnitine (C4:1) concentration. Increased abundance of bacteriocin ABC transporter and immunity protein-related genes was also highlighted in the TM5 birds when compared to the C diet. *Lactobacillus* strains can metabolize various monosaccharides and oligosaccharides into acetate (Gänzle, 2015) and are well known for their ability to produce both lantibiotics (Twomey et al., 2002) and bacteriocins (Hassan et al., 2020). Lantibiotics (such as lantacin B) are antimicrobial peptides (AMPs) whose production is regulated by cell-density-dependent, peptide pheromone-mediated quorum-sensing systems known in Gram-positive bacteria (Kleerebezem, 2004), while bacteriocins (such as Blp) are small AMPs that typically target organisms that are either closely related to or within the same species as the producer bacteria (Dawid et al., 2007). In particular, the sensing of own growth in lactic acid bacteria enables them to switch on bacteriocin production through bacteriocin ABC transporter at times when competition for nutrients intensifies (Mikkili et al., 2019; Eijsink et al., 2002), with the producing bacteria being protected from the bacteriocin effects by a specific immunity protein (Dawid et al., 2007). Therefore, the enrichment of these genes could have enabled better intra- and interspecies competition in the gut of these birds, thereby contributing to their improved growth performance. The cecal metabolome of the TM5-fed broilers shared some similarities with that of MIX5, in terms of increased dopamine and decreased 3-deoxyglucosone concentrations. Additionally, cecal tyramine concentration was higher in these birds compared with the C and HI10 groups. Interestingly, some SCFA-producing bacteria (e.g., *Enterococcus*) can decarboxylate tyrosine to the aromatic amine tyramine, which mediates peripheral serotonin production by EC cells and, in turn, modulates gastrointestinal motility and platelet function (Sugiyama et al., 2022). Therefore, the increased abundance of bacteriocin-related genes in the TM5 birds may reflect altered microbial competition, potentially influencing community structure in favor of selection for SCFAs-producing taxa and contributing to differences in biogenic amine production (such as dopamine and tyramine). Furthermore, the TM5-fed birds had higher quinoline-4-carboxylic acid and sphingomyelin C16:1 concentrations than the C and HI-based groups, along with lower concentrations of N-acetyl-histidine than the HI10 birds. Gut microbiota enzymes can convert dietary tryptophan into an array of quinoline derivatives that can act as quorum-sensing molecules (Heeb et al., 2011), while branched-chain sphingolipids, present in several anaerobic bacteria, can translocate into the host and maintain immune homeostasis by limiting inflammatory signaling (Bai et al., 2023). Furthermore, disruptions in histidine metabolism, leading to the production of histamine and N-acetyl-histidine, have recently been observed in broilers as an early indicator of the onset of necrotic enteritis (Gautam et al., 2025). Therefore, the improved growth performance of the TM5-fed birds may be related to an increased production of neurotransmitters and a decrease in metabolites indicative of perturbations in carbohydrate and amino acid metabolism. However, the increase in acylcarnitines relative to the C group may indicate impaired mitochondrial fatty acid oxidation. Nevertheless, the strong *Lactobacillus*-related microbiome signature could compensate for this source of reduced energy production. It should be noted that the decrease in sucrose-6-phosphate hydrolase and polysaccharide biosynthesis glycosyl transferase CpsN-related genes observed in the TM5 group seems to disagree with this signature, as *Lactobacillus* strains commonly produce sucrose-6-phosphate hydrolase (Gänzle, 2015) and are characterized by capsular polysaccharides (CPSs) exerting immunomodulatory activity (Li et al., 2022). However, as some pathogenic bacteria (such as *Streptococcus* and *Staphylococcus*) can also synthesize CPSs to evade phagocytosis, the reduced CPS synthesis is considered a positive outcome (Li et al., 2022).

In contrast to the positive gut microbiome signatures in TM5- and MIX5-fed broiler chickens, which allowed them to perform overall better than the other diets, the HI5 group showed a different pattern. The gut microbiota of these birds was associated with the SCFA-producing genus *Megasphaera* (Rychlik et al., 2020), which was positively correlated with 3-hydroxybutyric and 4-hydroxyphenylpyruvic acid metabolites. As butyrate-producing bacteria, this taxon can reasonably be involved in the so-called “ketone body-butyrate shuttle”, in which the gut microbiome supplies butyrate to the epithelium and the latter donates 3-hydroxybutyric acid to the former as an energy source in times of insufficient dietary carbohydrate availability (Satoh and Sasaki, 2024). Tyrosine aminotransferase transaminates tyrosine into 4‐hydroxyphenylpyruvate and glutamate, with the latter representing one of the main consumed metabolites by *Megasphaera* (Dell’Olio et al., 2024). As several SCFA-producing bacteria have previously been reported as characteristics of the gut microbiota of high-residual feed intake, low-feed efficiency birds (Liu et al., 2021), this gut microbiota signature may not have allowed the HI5 group to outperform the commercial feed. However, selected positive changes could have reasonably contributed to maintaining their growth performance similar to that of the C group. Indeed, their gut microbiome was associated with *Limosilactobacillus crispatus* (whose benefits have already been discussed for the TM5 birds) and with an increased abundance of *Lachnoclostridium* when compared to the C diet. Apart from being associated with high body weight, this SCFA-producing taxon has also been identified as a potential marker of improved meat quality in broiler chickens, characterized by increased muscle fiber diameter and decreased drip loss (Lei et al., 2022). Given that the HI5 diet was characterized by desirable meat quality traits (Biasato et al., 2025), this association warrants further investigation. Lastly, the increased levels of acetylcarnitines and 3-deoxyglucosone – previously outlined as negative metabolome signatures – may potentially be related to the selection of highly carbohydrate-fermenting taxa (such as *Limosilactobacillus crispatus* and *Lachnoclostridium*).

#### Gut histomorphometry

Unlike the gut microbiome signature discussed above, no specific histomorphological changes in the gut were observed in TM5- and MIX5-fed broiler chickens that could explain their improved growth performance. However, the preserved balance between neutral and acidic mucins - which were extensively correlated with bird weight, feed ingestion, and feed efficiency - may have contributed to growth outcomes. Indeed, the positive correlations between villus goblet cells secreting acidic sialylated and sulfated mucins, and bird feed intake indicate that a well-differentiated mucosal barrier is a prerequisite for sustaining high ingestion rates. Mucins provide essential lubrication that protects the epithelium from mechanical abrasion caused by digesta, and a compromised mucin layer can trigger feedback mechanisms to reduce feed intake to prevent mucosal damage (Montagne et al., 2003). Furthermore, the specific efficacy of acidic sulfated mucins in improving feed efficiency and supporting bird weight can be attributed to their chemical resistance to bacterial glycosidases. Unlike neutral mucins, acidic sulfated mucins form a rigid, electrostatic barrier that effectively prevents bacterial translocation and limits colonization by pathogenic microbiota (Deplancke and Gaskins, 2001). This resistance reduces the "maintenance energy" requirement of the gut, which would otherwise be spent on tissue repair and pathogen defense, thus allowing a greater proportion of metabolizable energy to be partitioned toward somatic growth (Uni et al., 2003).

### Gut health in low-performing broiler chickens

#### Gut microbiome

The significant differences between the HI10- and MIX10-fed birds (low-performing dietary treatments) and the C, and the TM5 and MIX5 groups (reference and high-performing dietary treatments, respectively), as revealed by the amplicon and shotgun metagenomics sequencing and metabolomics, are discussed below.

The cecal microbiota of the MIX10 broilers was significantly separated from that of the C group, with increased *l-Eubacterium* and decreased relative abundance of *Megasphaera* in MIX10. Both genera are SCFA producers (Rychlik et al., 2020), thus making it challenging to explain their different responses to the presence of dietary chitin. It is known that SCFA-producing bacteria can show a heterogeneous response to different carbohydrates, as substrate-driven gut microbiota changes have been linked to the different bacterial ability to degrade soluble or insoluble polysaccharides. However, different responses surprisingly yielded comparable SCFA production, indicating functional redundancy (Reichardt et al., 2018). *Ruminococcaceae,* able to ferment host-indigestible plant polysaccharides into SCFAs as well (Liu et al., 2021), were also more abundant in the MIX10 than the TM5 group. Interestingly, this family was negatively correlated with the ADFI and LW of the finisher phase, and the ADG and ADFI of the whole experimental trial, while *l-Eubacterium* was negatively correlated with the ADFI of the finisher phase. Overall, this agrees with the previous identification of *Ruminococcaceae* enrichment in the gut microbiota of high-residual feed intake, low-feed efficiency birds, confirming the complex influence of the SCFA-producing gut microbiota on feed efficiency in broilers (Liu et al., 2021). *Ruminococcaceae* were also positively correlated with concentrations of selected diacylglycerols and hexosylceramides, which are commonly identified in bacteria (Zhang et al., 2024). Therefore, aligned with our findings in the MIX5 group, it is more reasonable to hypothesize that these molecules are constituents of *Ruminococcaceae* rather than attributing an active role to their production. In parallel, *l-Eubacterium* was negatively correlated with the identification of selected diacylglycerols and hexosylceramides, N2-acetyl-l-ornithine, and orotic acid. This may suggest that this taxon is not characterized by the presence of those diacylglycerols and hexosylceramides, nor is it able to synthesize N2-acetyl-l-ornithine, in contrast to our findings for *Butyricicoccaceae* in the MIX5-fed birds. Importantly, the negative correlation between *l-Eubacterium* and N2-acetyl-l-ornithine and orotic acid could have contributed to the poor growth performance of the MIX10 broilers, as a consequence of their above-mentioned positive effects on gut health. Furthermore, the increase in acylcarnitines and N-acetyl-histidine compared with the MIX5 and C diets also needs to be considered in light of the above-discussed histidine metabolism disturbances and the negative influence on fatty acid oxidation.

The most prominent finding shared across all diets with a 10% inclusion level of insect meals is an association with *Alistipes*, which was positively correlated with fructose, fucose, and maltose, and with the urease accessory protein UreD, but negatively correlated with the PTS system and sucrose-specific IIB component-related genes. *Alistipes* strains have been identified as propionate producers in the chicken cecal microbiota (Polansky et al., 2015), but they are also characterized by the highest number of putrefaction pathways amongst commensal bacteria (Parker et al., 2020). As increasing inclusion levels of insect meals may reduce nutrient digestibility, undigested protein may accumulate in the ileum, leading to hindgut protein fermentation and, in turn, the formation of biogenic amines that may create a non-healthy gut environment (Biasato et al., 2023). Even if no significant increase in these toxic compounds was identified in the current study, the fact that *Alistipes* can contribute to histidine degradation and to indole and phenol production (Parker et al., 2020) warrants highlighting and deserves future attention. The PTS systems are carbohydrate transporters (with sucrose-specific IIA, IIB, or IIC components as protein domains) that are imperative for SCFA production and are less abundant in Bacteroidetes members, such as *Alistipes*, compared to Firmicutes (Mahowald et al., 2009). Ureases are nickel-dependent enzymes that catalyze urea hydrolysis to yield ammonia and bicarbonate, which, in turn, increase gut pH to values suitable for the survival of pathogenic bacteria (Masetti et al., 2020). *Alistipes* strains are generally urease-negative (Parker et al., 2020), making it challenging to explain their positive relationship with the urease accessory protein UreD. However, increased abundance of *Alistipes* has recently been detected in patients with hyperuricemia, potentially due to its effect on purine metabolism by upregulating xanthine dehydrogenase, which degrades purine to uric acid (Shirvani-Rad et al., 2023). As some bacteria (e.g., *Enterobacteriaceae*) possess allantoinase activity, another enzyme in purine metabolism that degrades uric acid to urea (Guo et al., 2016), we can hypothesize disturbances in purine metabolism characterized by elevated uric acid levels, selection of allantoinase-producing bacteria, and upregulation of urease enzymes. Lastly, as *Alistipes* has recently been identified as a biomarker of high-sugar diets in the gut microbiota of mice (Tan et al., 2022), its positive relationship with fructose, fucose, and maltose seems reasonable. Diets containing 10% insect meals also showed lower percentages of phage lysozyme and phage integrase family protein-related genes compared with the C group. Phages targeting specific bacteria by producing various enzymes, such as lysozyme and integrase, can hydrolyze the peptidoglycan layer of the bacterial cell wall or facilitate DNA recombination between the phage and the bacterial attachment site, thereby increasing phage survival in the host cell (Mahmud et al., 2024). Therefore, such a reduction in these enzymes may have reasonably contributed to the reduced growth performance of the HI10- and MIX10-fed birds. Furthermore, the cecal metabolome of the HI10 broilers was characterized by a decrease in positive metabolites (such as dopamine and tyramine) and an increase in detrimental metabolites (such as N-acetyl-histidine, 3-deoxyglucosone, and hexanoylcarnitine).

In contrast to the above-discussed negative gut microbiome signature in MIX10- and HI10-fed broilers, which led them to perform overall worse than the other diets, the TM10 group showed different patterns. The gut microbiota of these birds was associated with *Eubacterium* and *Tyzzerella*, with decreased *Campylobacter jejuni* and increased *Limosilactobacillus crispatus* abundance compared to the C diet. Given the above-mentioned negative influence of *Ruminococcaceae* members on feed efficiency in broilers (Liu et al., 2021), the *Eubacterium* association may not have allowed the TM10 group to outperform the commercial feed. However, at the same time, other positive changes could have reasonably contributed to maintaining their growth performance similar to that of the C-fed broilers. The benefits of *Limosilactobacillus crispatus* have already been discussed for the TM5 group. *Tyzzerella* has been reported to increase after probiotic supplementation in poultry (Gao et al., 2017), and is also positively associated with Vh and Vh/Cd (Heak et al., 2017; Wiersema et al., 2021). The decrease in *Campylobacter jejuni* is the most striking finding especially because it was positively correlated with the multidrug resistance RND efflux system inner membrane transporter MexD and MexF, macrolide-specific efflux protein MacA, penicillin binding protein 2 (PBP2), multidrug efflux transporter major facilitator superfamily MFS TC 2A1, broad specificity multidrug efflux pump YkkD, and beta-lactamase related genes. Small multidrug resistance (SMR) proteins, such as resistance/nodulation/cell division family (RND) and the major facilitator superfamily (MFS) of the multidrug resistance efflux systems/transporters, are involved in the development of intrinsic and acquired resistance of bacteria to various antimicrobials (Bay et al., 2008), as well as efflux pumps specific to macrolides (MacA and MacB; Guérin et al., 2020). In parallel, penicillin-binding proteins (PBPs) support the normal biosynthesis of the peptidoglycan layer for bacterial survival and growth, while β-lactamases presumably confer pathogens' resistance to β-lactam antibiotics (Choi et al., 2024). Most of these proteins have previously been identified in *Campylobacter jejuni* (Bay et al., 2008; Guérin et al., 2020; Choi et al., 2024), which is the leading cause of human bacterial diarrheal disease related to the handling and consumption of poultry meat products (Kelbert et al., 2025). Biasato et al. (2023) recently reported that insect-based diets for monogastric species may reduce pathogen levels in their gut microbiota, as a consequence of the synergistic activity of their specific nutraceutical components (i.e., lauric acid, chitin, and antimicrobial peptides) that contribute to their antimicrobial properties. However, the current research represents the first report of TM-related antimicrobial activity in the intestinal tract of chickens, also shedding light on its potential to reduce antimicrobial resistance. Lastly, the cecal metabolome of the TM10-fed broilers showed higher levels of tyramine and sphingomyelin C16:1 than with the C and HI10 diets, suggesting enhanced immune homeostasis and increased neurotransmitter production.

#### Gut histomorphometry

Along with the above-discussed gut microbiome signature, the MIX10-fed birds showed thinner mucosal and muscular layers than the C and HI5 groups, and tended to display shorter villi than the C group. Increasing the inclusion levels of insect meals in broiler diets has been linked to worsening morphological features of the gut mucosa, thereby reducing nutrient digestibility (Biasato et al., 2023). While changes in the intestinal muscular layer have not been previously reported in broilers fed insect meals, the decreased abundance of some SCFA-producing bacteria in the gut of the MIX10 group might have impacted its thinning, as increasing butyrate proportions and SCFA concentrations seem to stimulate cecal muscle contraction in laying hens (Yosi et al., 2022). The significant positive correlation observed between jejunal Vh and ADG, alongside the inverse relationship with FCR, substantiates a direct link between mucosal architecture and nutrient-acquisition efficiency, thereby explaining the poor growth performance observed in MIX10-fed birds. Indeed, increased Vh expands the absorptive surface area, enhancing the transport of monosaccharides and AA across the brush border membrane, which is critical for maximizing growth efficiency during the finisher phase (Awad et al., 2009). As *Ruminococcaceae* members (such as *Ruminococcus gnavus*) can degrade mucins (Crost et al., 2016), their increased abundance in the gut of the MIX10 group may be responsible for the reduction in villi neutral mucins, leading to an imbalance between neutral and acidic mucins and, consequently, contributing to poor growth outcomes. A similar scenario was also observed in the HI10 group, where the decrease in villi neutral mucins could be related to the high concentrations of 3-deoxyglucosone, which reacts with amino groups, particularly abundant in mucins. However, the fact that the HI5 group displayed the same results makes the mucin changes less relevant than the gut microbiome in shaping bird growth outcomes. The same consideration applies to the widened villi observed in both HI groups, which can be attributed to the high lauric acid content of the HI meal. Indeed, its readily absorbable energy and antimicrobial benefits may have facilitated the lateral expansion and maturation of villus enterocytes without inducing the proliferative crypt turnover (Al Anas et al., 2024; Kierończyk et al., 2022). As jejunal inflammation was unaffected by dietary treatments and negatively correlated with bird weight gain, it can be excluded as a factor in the modulation of growth performance. Indeed, inflammatory responses trigger the release of pro-inflammatory cytokines, which divert nutrients away from skeletal muscle accretion towards hepatic acute-phase protein synthesis and immune cell proliferation, thereby depressing growth performance (Klasing, 2007).

## Conclusions

High-performing birds (MIX5 and TM5) exhibited cecal microbiomes enriched in SCFA-producing taxa, including *Veillonellaceae, Butyricicoccus*, and *Limosilactobacillus crispatus*, which were associated with improved feed efficiency and favorable shifts in the cecal metabolome. These changes included increased concentrations of bioactive metabolites, including dopamine and tyramine, as well as malic and orotic acids, which are linked to the microbiota-gut-brain axis, energy metabolism, and gut health. In particular, the MIX5 diet promoted a unique microbiome signature characterized by a high abundance of *Butyricicoccaceae*, which correlated with improved lipid and amino acid metabolism, while the TM5 diet supported bacteriocin-producing *Lactobacillus* strains and demonstrated potential antimicrobial and competitive-exclusion effects. Both these groups showed reductions in metabolites indicative of metabolic dysfunctions, such as 3-deoxyglucosone and hexanoylcarnitine. While no single histomorphology trait explained the superior performance of these groups, the preservation of gut mucin balance was strongly associated with higher feed intake, improved feed efficiency, and greater body weight, highlighting the importance of an intact, well-differentiated mucosal barrier.

Conversely, low-performing birds (MIX10 and HI10) displayed gut microbiome signatures dominated by *Ruminococcaceae, Alistipes*, and *l-Eubacterium*, taxa associated with impaired feed efficiency and altered purine metabolism. These birds also presented increased levels of metabolites associated with mitochondrial dysfunction and compromised gut health, such as acylcarnitines and N-acetyl-histidine. These microbial shifts were accompanied, particularly in the MIX10-fed birds, by compromised gut morphology, including thinner mucosal and muscular layers. They also showed reduced Vh, which was positively correlated with growth rate and negatively correlated with feed efficiency. The increased abundance of mucin-degrading bacteria (in the MIX10 group) or 3-deoxyglucosone concentrations (in the HI10-fed broilers) likely contributed to altered villus mucin composition, reinforcing poor growth outcomes. In contrast, jejunal inflammation was unaffected by insect meal utilization, suggesting that microbiome-mucosal interactions, rather than inflammatory processes, primarily drove growth modulation.

Among the high-insect meal groups, TM10 maintained a gut microbiome that supported stable performance. This was likely due to the presence of beneficial taxa like *Limosilactobacillus crispatus* and *Tyzzerella*, alongside a marked reduction in *Campylobacter jejuni* and associated antimicrobial resistance genes, suggesting an added benefit of TM in enhancing gut microbial safety and reducing antimicrobial resistance.

Overall, the results of the present study underscore the complexity of host-gut microbiome-diet interactions and identify specific microbial and mucosal features as potential biomarkers of optimal performance in broilers fed insect-based diets.

## Ethics approval and consent to participate

The experimental protocol was designed in accordance with the current European Directive (2010/63/EU) on the care and protection of animals used for scientific purposes and was approved by the Ethical Committee of the University of Turin (Italy) (Protocol No. 15735).

## CRediT authorship contribution statement

**Ilaria Biasato:** Writing – original draft, Validation, Supervision, Methodology, Data curation, Conceptualization. **Talal Hassan:** Writing – original draft, Software, Methodology, Formal analysis, Data curation, Conceptualization. **Davide Buzzanca:** Writing – original draft, Software, Methodology, Formal analysis, Data curation. **Stefano Bagatella:** Writing – review & editing, Methodology, Formal analysis. **Achille Schiavone:** Writing – review & editing, Supervision. **Maria Teresa Capucchio:** Writing – review & editing, Supervision, Methodology, Formal analysis. **Laura Gasco:** Writing – review & editing, Supervision, Funding acquisition. **Ákos Kenéz:** Writing – original draft, Validation, Software, Methodology, Formal analysis, Data curation, Conceptualization. **Ilario Ferrocino:** Writing – review & editing, Validation, Supervision, Methodology, Conceptualization.

## Disclosures

The authors declare that they have no known competing financial interests or personal relationships that could have appeared to influence the work reported in this paper.

## Appendix Supplementary materials

Image, application 1

## Footnotes

Supplementary material associated with this article can be found, in the online version, at doi:10.1016/j.psj.2026.106966.
